# Specific Non-Local Interactions Are Not Necessary for Recovering Native Protein Dynamics

**DOI:** 10.1371/journal.pone.0091347

**Published:** 2014-03-13

**Authors:** Bhaskar Dasgupta, Kota Kasahara, Narutoshi Kamiya, Haruki Nakamura, Akira R. Kinjo

**Affiliations:** Institute for Protein Research, Osaka University, Suita, Osaka, Japan; Wake Forest University, United States of America

## Abstract

The elastic network model (ENM) is a widely used method to study native protein dynamics by normal mode analysis (NMA). In ENM we need information about all pairwise distances, and the distance between contacting atoms is restrained to the native value. Therefore ENM requires *O*(*N^2^*) information to realize its dynamics for a protein consisting of *N* amino acid residues. To see if (or to what extent) such a large amount of specific structural information is required to realize native protein dynamics, here we introduce a novel model based on only *O*(*N*) restraints. This model, named the ‘contact number diffusion’ model (CND), includes specific distance restraints for only local (along the amino acid sequence) atom pairs, and semi-specific non-local restraints imposed on each atom, rather than atom pairs. The semi-specific non-local restraints are defined in terms of the non-local contact numbers of atoms. The CND model exhibits the dynamic characteristics comparable to ENM and more correlated with the explicit-solvent molecular dynamics simulation than ENM. Moreover, unrealistic surface fluctuations often observed in ENM were suppressed in CND. On the other hand, in some ligand-bound structures CND showed larger fluctuations of buried protein atoms interacting with the ligand compared to ENM. In addition, fluctuations from CND and ENM show comparable correlations with the experimental B-factor. Although there are some indications of the importance of some specific non-local interactions, the semi-specific non-local interactions are mostly sufficient for reproducing the native protein dynamics.

## Introduction

The biological function of a protein cannot be completely understood unless roles of the structure and its dynamics are characterized. One method to obtain dynamic characteristic of a protein around its native structure is the normal mode analysis (NMA) [1], [2]. Although molecular dynamics (MD) simulations give more accurate dynamic pictures, NMA has its own advantages [3], such as lower computational cost and analytically obtained normal modes that capture native protein dynamics reasonably accurately.

The elastic network model (ENM) is currently the most popular model for NMA [4]. In ENM the protein structure is modeled as a set of atoms and each contacting pair of atoms are connected by a harmonic spring. Moreover, the equilibrium length of such a spring is set to the distance between the corresponding atom pairs in the native structure. Therefore, the experimentally obtained structure is guaranteed to be at the global energy minimum. Because of this favorable property, ENM has been used extensively [5]–[11].

In ENM the dynamics of a protein is characterized by using all pairwise native distances. That is, *O*(*N^2^*) information for a *N*-residue polypeptide. Therefore, from a “sequence-determines-structure-(via-dynamics)determines-function” view one may argue that a protein sequence needs to include all pairwise distance information to express its function. This may be a prohibitively large amount of information to be embedded in the protein sequence. It is interesting to see whether a protein structure actually needs this much information to realize its native dynamics.

One of the drawbacks of ENM is that it does not include any protein-solvent interactions. Therefore, the surface atoms are involved in a less number of interactions than the core atoms. Such atomic packing may cause unrealistic fluctuations of the surface atoms. We observed significantly high surface fluctuations in our previous study [12]. The unrealistic fluctuations of surface atoms were also observed by Wako and Endo using only dihedral angles as variables rather than all Cartesian coordinates [13]. If we consider protein-solvent interactions then the surface atoms would fluctuate to a lesser extent (due to solvent-dampening of the fluctuations). Therefore, it is desirable to have an implicit-solvent model that guarantees the native structure to be at a local energy minimum.

In addition, there is a conceptual drawback in ENM that a protein molecule is treated as a purely mechanical system composed of atoms and springs. In this picture, some basic physicochemical properties of the protein are not explicitly taken into account. For example, ENM treats chemical bonds and physical contacts indiscriminately so that the polypeptide structure is of no importance, and the well-known “hydrophobic in, hydrophilic out” principle of globular proteins is absent. While such treatment of ENM comprises the simplicity and beauty of the model, it also makes it difficult to connect the physics of dynamics with the biology of sequence.

To address the above issues in ENM we introduce here a new model, named the contact number diffusion model (CND). The contact number of an atom is the number of atoms that surround the given atom in the native structure, and it is closely related to the hydrophobicity of amino acid residues [14], [15]. In CND, we model the protein structure as an autonomous system of local interactions and non-local contact numbers that are biased to the native structure. Here we say two atoms are locally interacting if the corresponding residues are separated within a certain number (window size) of residues along the sequence. Atom pairs that are not locally interacting are called non-local pairs. The autonomous term of the local interactions tends to break the local structures. On the other hand, that of the non-local contact numbers tends to uniform contact numbers by their diffusion along the polypeptide chain. The tendency for uniform contact numbers corresponds to the autonomous behavior (that is, without native bias) of the model that makes the polypeptide chain to form any random structures in which no residue is particularly buried or exposed. In addition to these autonomous behaviors of the “generic polypeptide” chain, a natural protein with a particular amino acid sequence has a specific bias toward the native structure under the physiological condition. Identifying precise interactions comprising the native bias is a complicated matter. In our model, such bias toward the native structure is imposed through the Lagrange multipliers to constrain local contacts and non-local contact numbers to the native values. The non-local interactions in the CND are said to be “semi-specific” because they are biased to the native structure only in terms of the contact number, which is defined for each atom instead of each pair of atoms.

In CND the interaction network of protein atoms consists of non-local contact numbers and local contacts. Therefore, the requirement of *O*(*N^2^*) restraints in ENM is reduced to only *O*(*N*) restraints in CND. We show in the following that such a reduced set of restraints is sufficient to reproduce native protein dynamics. Moreover, due to the contact number restraints in CND and its multi-body nature, the fluctuations of surface atoms are lower compared to ENM. Thus the drawback of ENM regarding unrealistic surface fluctuations is reduced in CND. Furthermore, since we separate local interactions from non-local ones, the chain structure of the protein is more explicit; and since non-local interactions are treated in terms of contact numbers rather than pairwise contacts and the contact number is dual to hydrophobicity [14], [15], the CND model can be more easily correlated with the properties of amino acid sequence.

We compared the characteristics of the normal modes obtained from CND and ENM. Thus obtained normal modes were evaluated in comparison with (1) explicit solvent MD simulation, (2) apo-holo conformational change, and (3) crystallographic B-factor. We observed that CND correlated better with MD simulation than ENM. CND and ENM fit equally well to the apo-holo conformational changes of 13 pairs of proteins [13]. In many cases CND and ENM were comparable in terms of correlation between atomic mean-square fluctuations (MSF) and experimentally observed B-factor. In addition, we found that the normal modes obtained from CND are more collective than those from ENM.

## Theory

### 1) Normal Mode Analysis (NMA)

Let a protein molecule consist of *N* atoms with coordinates **r**_i_ = (x_i_, y_i_, z_i_)^T^ = (x_i,1_, x_i,2_, x_i,3_)^T^, where *i* = 1,…,*N* and superscript ‘T’ indicates transpose operation. We remark the native structure with the superscript ‘0′ in the following sections, e.g. **r**_i_^0^ indicates the native coordinate of atom *i*. In NMA, the potential energy of the native structure (U({**r**_i_^0^})) is assumed to be at a local minimum, and therefore, the potential energy at any instance (*t*) can be approximated as
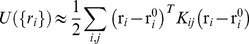
(1)where constant and higher order terms are neglected and



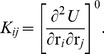
(2)Based on this linearized potential function, the equation of motion is given as
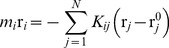
(3)where *m_i_* is the mass of the atom *i* and 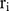 denotes the second-order derivative of coordinate with respect to time. By introducing the generalized mass-weighted coordinates 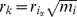, where 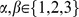 and 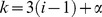, 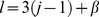, the elements of the mass-weighted Hessian H are represented as,



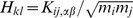
(4)Solving the above equation of motion (eq. 3, 4) reduces to solving the eigenvalue problem in generalized mass-weighted coordinates,
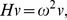
(5)the result of which is a set of normal modes, i.e., eigenvalues *ω_k_^2^* and the corresponding eigenvectors *ν_k_* (*k* = 1,…,*3N*).

### 2) Contact Number Diffusion (CND) Model

In CND two atoms (*i* and *j*) are defined to be locally interacting when the corresponding residues are separated by at most *w* residues along the chain. To implement this we introduce an *N*x*N* matrix *θ* the element of which is 1 if two atoms are locally interacting and 0 otherwise.

One of the most essential ingredients of CND is non-local contact number (*n_i_*) defined as
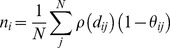
(6)where *ρ* is a non-negative monotonically decreasing function of the distance (*d_ij_*) between atoms *i* and *j*. This definition of contact number is a slight modification of those used in previous studies [16]–[18]. In the present study the functional form of *ρ(d_ij_)* is,
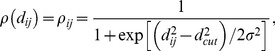
(7)where dcut is a cutoff distance (5 Å in the current study) and σ determines the steepness of the sigmoidal function.

The energy function of CND is given as
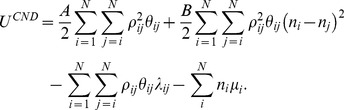
(8)


The first two terms in the right-hand side of this equation model the autonomous behavior of the system and the last two terms bias the autonomous system to the native structure through the Lagrange multipliers *λ_ij_* and *μ_i_*. The first term on the right hand side includes all local pairwise distances and destabilizes the local structure. The second term on the right hand side penalizes heterogeneity of the contact numbers along the polypeptide chain. Therefore, the autonomous behavior of the system tends to unfold the structure. The constants *A* and *B* are free positive parameters.

To obtain the native restraints we need to determine the values of *λ_ij_* and *μ_i_*, which is done by setting the Jacobian of the above energy function
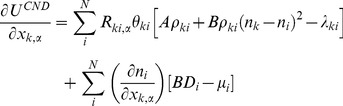
(9)to zero at the native structure. Here we define



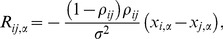
(10)

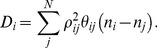
(11)


This *D_i_* term can be interpreted as a diffusion of contact numbers along the polypeptide chain. That is, if *n_i_* in the summation is large compared to its neighboring atoms, *D_i_* is large and the atom *i* tends to move to the direction where its contact number *n_i_* will decrease (or the neighboring atoms will diffuse away). A solution to 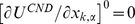 is
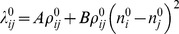
(12A)




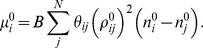
(12B)


Here equation (12A) is meaningful only for local pairs.

It is worth explaining the behavior of the model in terms of the force (Eq. 9). As for the local pairs (the first term on the right hand side of Eq. 9), the term 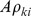 originating from the first term of Eq. 8 tends to break the local structure. This tendency is strengthened by the term 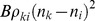 originating from the second term of Eq. 8 especially if two atoms have very different contact numbers. That is, a local pair of atoms, one with a large contact number and the other with a small contact number, will strongly repel each other. If both atoms have similar contact numbers, whether large or small, the repulsion is not so strong. Nevertheless, this autonomous behavior is corrected by the native constraint *λ_ki_^0^* (c.f. Eq. 12A), which represents the intrinsic tendency for specific local structures of the given protein. The second term on the right hand side of Eq. 9 contains the diffusion term *D_i_* so that an atom with a relatively large contact number (compared to its local neighbors) tends to move to a less crowded region in space whereas an atom with a relatively small contact number to a more crowded region so that the contact number tends to be uniform along the polypeptide chain. Again, this autonomous behavior is corrected by the native constraint μ*_i_^0^* (c.f. Eq. 12B), which represents the intrinsic tendency of atomic burial (or hydrophobicity) of the native protein structure. Note that restraining the contact number (with protein atoms) implicitly restrains the number of contacts with solvent atoms to the value that is favored in the native structure. In this manner, the diffusion term *D_i_* together with the native constraint term μ*_i_^0^* models protein-solvent interactions implicitly. In summary, the autonomous terms, representing the default behavior of a feature-less generic polypeptide chain, tend to break local and non-local structures, the former by repulsive forces between local pairs and the latter by uniforming contact numbers; the constraint terms correct this autonomous behavior by counterbalancing it with the opposing forces produced at the native structure.

Now that we have determined the multipliers *λ_ij_^0^* and μ*_i_^0^*, we can obtain the Hessian at the native condition. The Hessian can be written as a 3×3 matrix each element of which is an *N*×*N* matrix. Each such block is defined as,
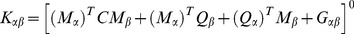
(13)where we defined the following matrices,



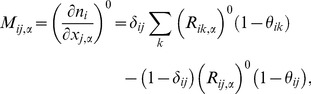
(14A)

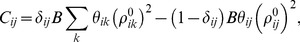
(14B)




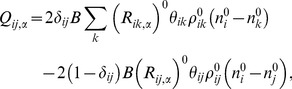
(14C)




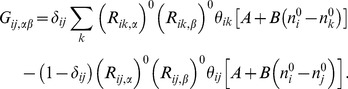
(14D)


### 3) Elastic Network Model (ENM)

The elastic network model describes a protein structure as a set of atoms interconnected by a network of Hookean springs [4]. The potential energy function for the ENM is given by,
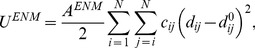
(18)where *c_ij_* are the spring constants and *A^ENM^* is a phenomological constant that we set to unity. The Jacobian of *U^ENM^* is given as,
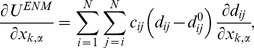
(19)and the mass-unweighted Hessian at the native configuration is given as,



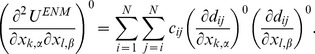
(20)An *N*×*N* block of the mass-unweighted Hessian matrix is given as 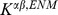, the (*i,j*) element of which is given as,
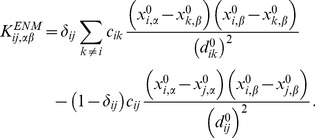
(21)


In the present study we defined
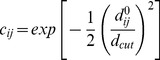
(22)where *d_cut_* is 5 Å, in accordance with previous works [12], [13].

In comparison with the contact number diffusion model, a model analogous to ENM can be formulated. By setting 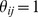 for all *i, j* in eq. (8), we have
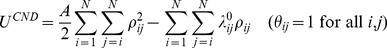
(23)where 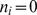 for all *i* (c.f. Eq. 6) and 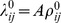 (c.f. Eq. 12A). Substituting these *λ_ij_^0^* values in eq. (23) under native condition we get



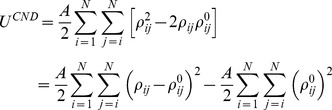
(24)The second term in the right-hand side of eq. (24) is a constant, which only shifts the absolute value of the energy. The first term 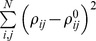 is analogous to the standard ENM potential energy function (eq. (18)). This indicates that, in essence, ENM is a special case of CND where the window size is sufficiently large.

## Results

### 1) Low-frequency Modes are More Dominant in CND than in ENM and MD

**Figure 1:**
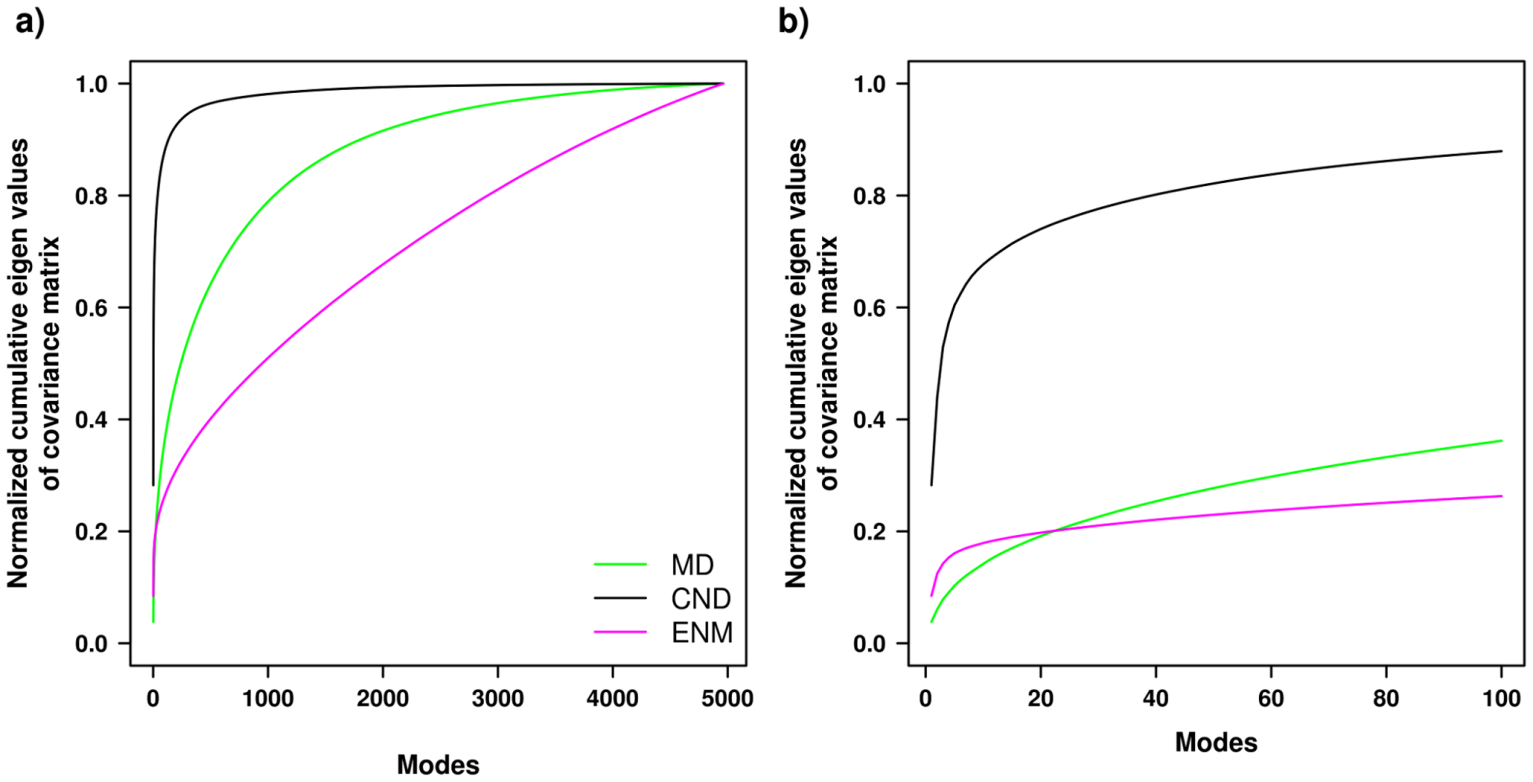
Dominance of low-frequency modes. Cumulative eigenvalues of covariance matrix normalized by sum of eigenvalues obtained from CND, ENM and MD simulation for ADK^A^. a) The trend over all the modes, and b) the trend over 100 lowest-frequency modes.

In normal mode analysis low-frequency modes are often analyzed to gain insight about collective motions of a protein. We compared the distribution of eigenvalues of the covariance matrices obtained from CND and ENM as well as a MD trajectory of ligand-free adenylate kinase from *Escherichia coli* (referred to as ADK^A^ in the following) (Figure 1). Note that the covariance matrices were analyzed instead of Hessian matrices so that the MD trajectory can be compared with normal modes. The CND normal modes saturated more rapidly than ENM normal modes and MD principal modes, whereas ENM normal modes saturated more slowly than MD principal modes (Figure 1a). The first 50 low-frequency modes in CND accounted for the 82% of the overall variance (corresponding values for ENM and MD were 23% and 28%, respectively; Figure 1b). In summary, the first few collective low-frequency modes of CND largely dominated the overall dynamics compared to ENM and even MD.

### 2) Dynamics from CND Model are Correlated with MD Simulation

**Figure 2:**
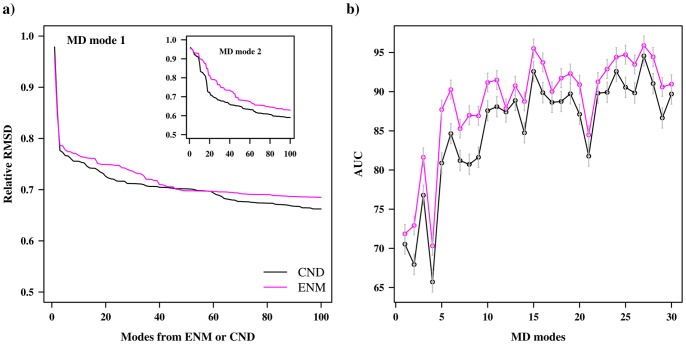
Fitting of CND and ENM normal modes to MD principal modes. Least-square fitting of 1^st^ and 2^nd^ principal modes (in (a)) by linear combinations of 100 lowest-frequency normal modes obtained from CND and ENM. The normal modes were taken cumulatively to fit to the MD data (by varying *N_m_*, see methods). The relative RMSD is defined in the section 5 of Materials and Methods. b) The area under the curve (AUC) obtained from the fitting of first 30 principal modes. The AUC obtained for each principal mode was obtained from the sum of the relative RMSD over 100 normal modes. The error bars were calculated by bootstrapping over all the AUC values. A larger AUC for the higher principal modes results from poorer fitting by the normal modes. This indicates that the lowest 100 normal modes less efficiently fit higher principal modes and more efficiently fit lower principal modes.

To compare the dynamics obtained from CND and ENM to MD simulation more concretely, we compared CND and ENM normal modes (100 lowest-frequency normal modes) to the MD principal modes (30 lowest-frequency modes) by cumulative least-square fitting (see Materials and Methods, Figure 2). Trivially, more normal modes would fit better to a principal mode, as demonstrated by a monotonic decrease of relative RMSD with increasing number of normal modes (Figure 2a). The areas under the curve (AUC) obtained from the cumulative fitting of the principal MD modes by ENM and CND normal modes were compared (Figure 2b). We observed that the AUCs of the ENM modes were greater than those of CND modes for majority of the principal modes. This indicates that the normal modes of CND better capture principal modes of the MD simulation.

**Figure 3:**
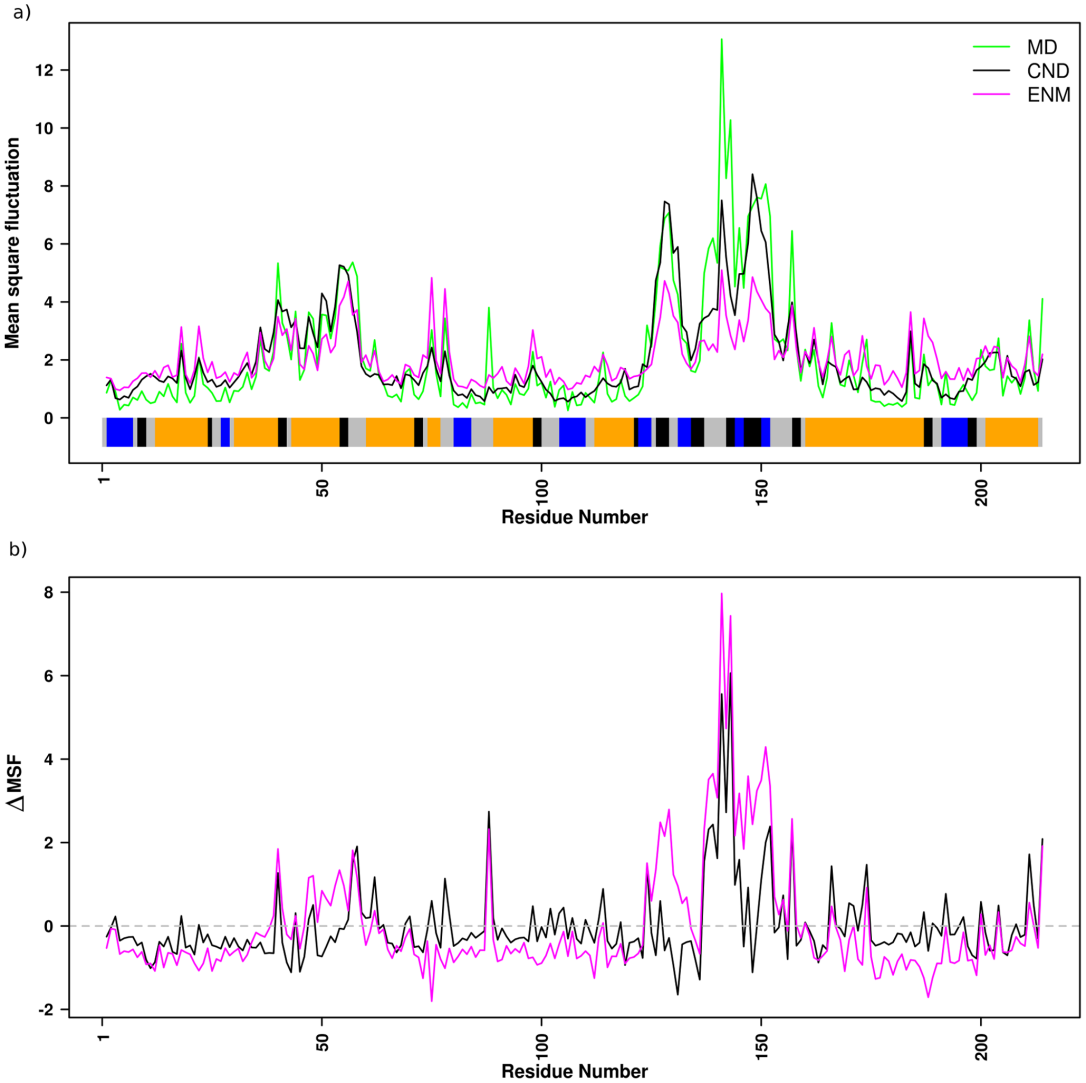
Mean square fluctuation (MSF) of CND, ENM and MD. a) MSF of residues averaged over atoms obtained from CND, ENM normal modes and MD simulation for ADK^A^. The MSF from the normal mode analyses were scaled so that the average MSF over all residues were equal. The bottom panel indicates color-coded DSSP secondary structures [65], where β-strand are in blue, helices are in orange, turns are in black and others are in gray. b) Difference of MSF between MD and CND (or ENM). The horizontal gray line indicates identical MSF from MD and NMA given for visual guidance.

We also compared mean square fluctuations (MSFs) obtained from the CND and ENM models to those of the MD simulation (Figure 3). We observed that the correlation coefficient between MSFs of CND and MD was 0.89, which was higher than that between ENM and MD (0.83). A closer inspection of the MSFs revealed that the fluctuation of CND especially better correlated with MD around residues 130 to 150. These observations can be further verified from Figure 3b where the differences of NMA-based MSF from MD-based MSF are plotted.

### 3) Normal Modes Obtained from CND and ENM fit Experimental Conformational Changes

**Figure 4:**
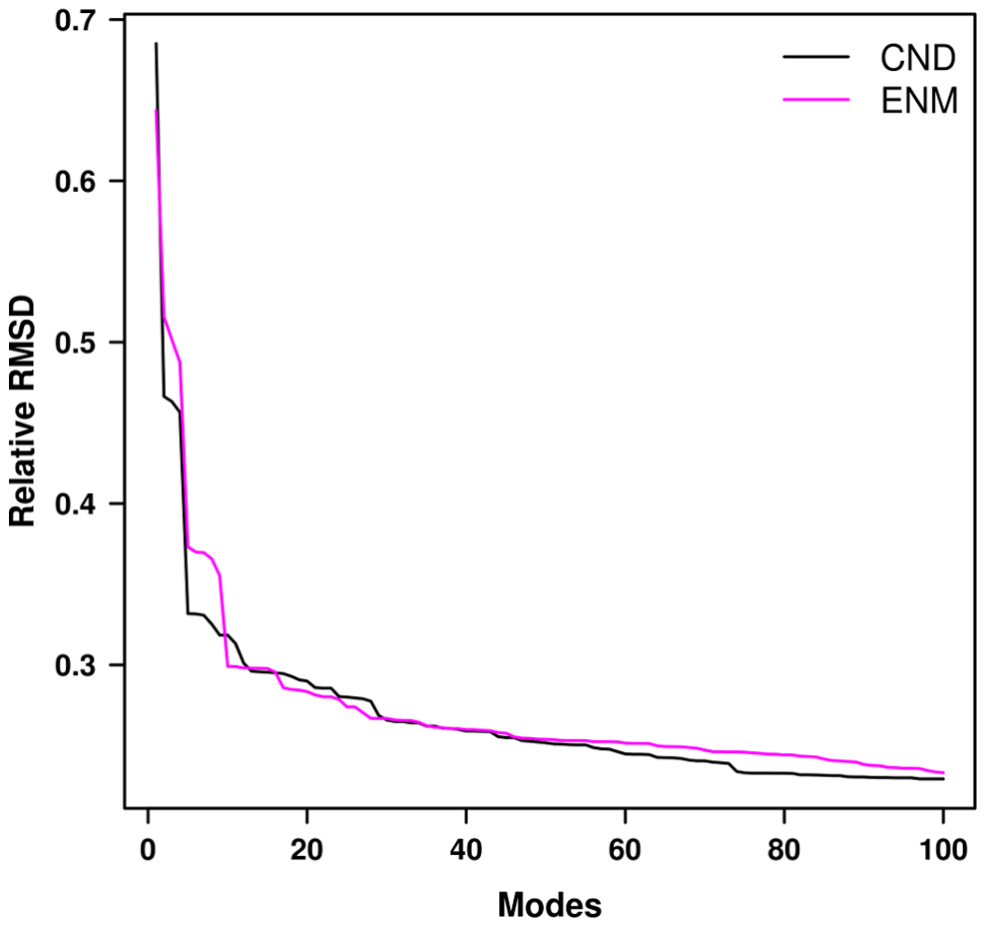
Fitting of CND and ENM normal modes to a conformational change. The relative RMSD obtained from the least-square fitting of the conformational change between 4AKE (apo) and 1AKE (holo) by the 100 lowest frequency normal modes of apo structure.

**Figure 5:**
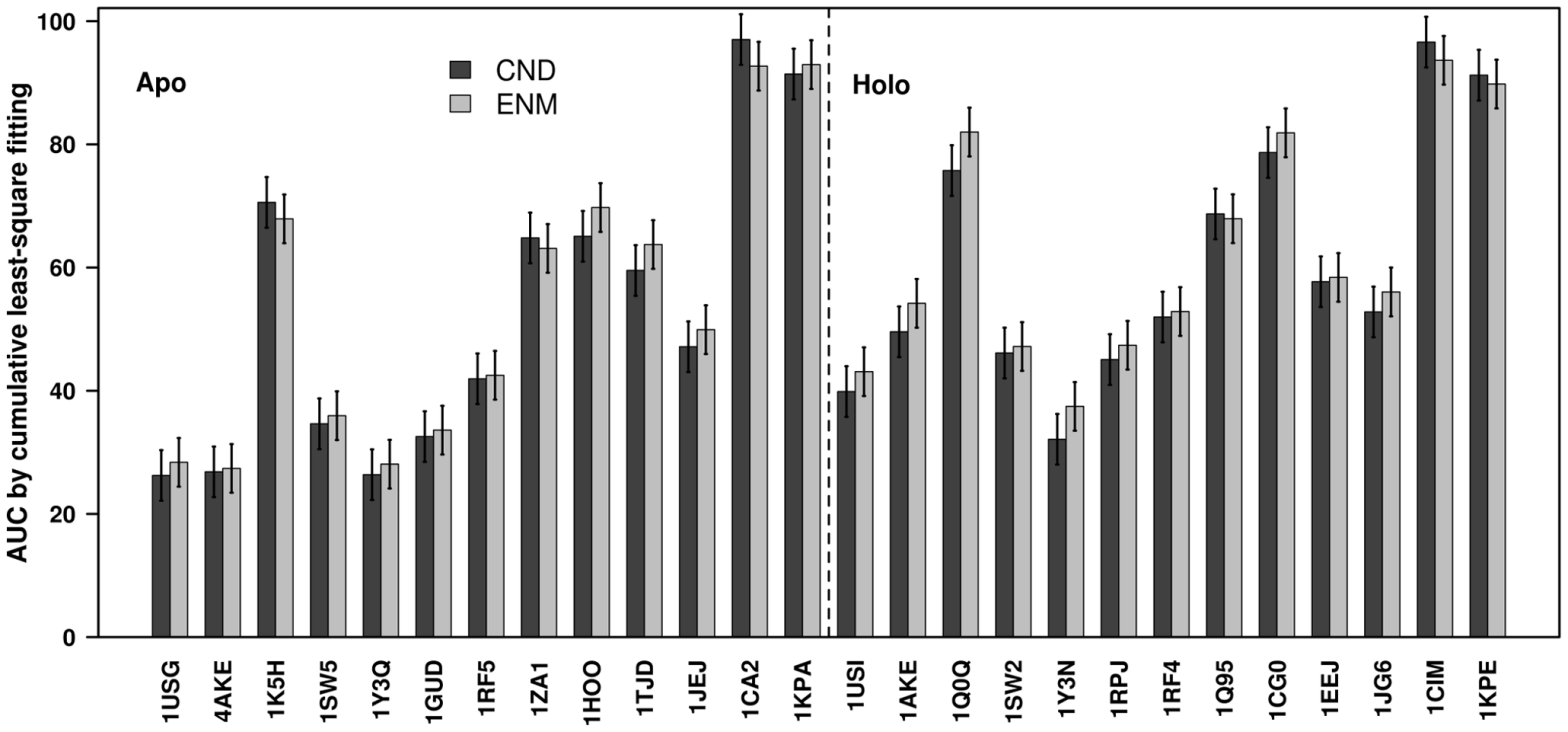
Comparison of fitting of CND and ENM normal modes to conformational changes. The area under the curve (AUC) obtained by fitting conformational change between apo and holo structure by a cumulative linear combination of 100 lowest-frequency normal modes (see methods and equation (28)). The apo and holo structures were separated by a dotted line.

One of the advantages of studying the normal modes obtained from ENM is that these modes often reproduce a conformational change between two conformations of a protein [11], [13]. In general conformational changes can be studied by comparing apo-holo pairs, and Table 1 lists 13 such pairs used in the current study. We compared the performances of the CND and ENM models in the same way as we did for the comparison with the MD simulation. That is, we fitted the conformational changes by linear combinations of up to the first 100 low-frequency normal modes obtained from CND or ENM (Figure 4). We observed that in all the pairs fitting by CND and ENM are comparable (Figure 5).

**Table 1:** Dataset of apo and holo structures [30]–[49], corresponding root-mean square deviation (RMSD) (all-atom superimposition^a^) and previous studies on the data set^b^.

Apo			Holo			RMSD(Å)	Large-scale domainmotion [50]	Side-chainflexibility [51]	Prediction of holoconformation [52]
PDB ID^c^	Chain ID	Resolution (Å)	PDB ID	Chain ID	Resolution (Å)				
1USG	A	1.53	1USI	A	1.80	7.32	o^d^	x	o
4AKE	A	2.20	1AKE	A	2.00	7.19	o	o	x
1SW5	A	1.80	1SW2	A	2.10	5.29	o	x	o
1K5H	A	2.50	1Q0Q	A	1.90	5.28	o	x	x
1Y3Q	A	1.90	1Y3N	A	1.60	4.91	o	x	o
1GUD	A	1.71	1RPJ	A	1.80	4.72	o	x	o
1RF5	A	2.30	1RF4	A	2.20	3.92	o	x	o
1ZA1	A	2.20	1Q95	A	2.46	2.57	o	x	x
1HOO	B	2.30	1CG0	A	2.50	2.51	o	x	x
1TJD	A	2.50	1EEJ	B	1.90	2.38	o	x	x
1JEJ	A	2.50	1JG6	A	1.90	2.31	o	x	o
1CA2	A	2.00	1CIM	A	2.10	0.64	x	o	x
1KPA	B	2.00	1KPE	B	1.80	0.53	x	o	x

a RMSD were obtained by superimposition of apo structure to holo structure [66].

b Previously the data set were used in predicting conformational change between apo and holo conformation by a linear combination normal modes obtained under ENM by Wako and Endo [13]. This data set was part of other studies as referred in last three columns [50]–[52]. The large scale domain motion was studied by Brylinski and Skolnick [50] for almost all the pair of structures. Side-chain flexibility between apo and holo structures of few pairs in the present data set was studied by Najmanovich *et al* [51]. A few pair of structures was part of docking studies to predict holo conformation from apo conformation by Seeliger and De Groot [52].

c In the current study a protein structure is often referred by its 4-letter PDB identifier [57].

d ‘o’ and ‘x’ indicates (in last three columns) the whether or not the corresponding pair was previously included in a study cited at the column header.

The conformational changes can be fitted by using normal modes based on either holo or apo structures. Previous studies have shown that ENM based on holo structures, rather than apo structures, can better fit conformational changes [11], [13]. This is indeed confirmed in the present study (Table 2). But, we also find that the same trend applies to CND. Hence, CND is comparable to ENM in this respect.

**Table 2:** Coverage of conformational change^a^ obtained from the least-square fit to the conformational change between apo and holo by normal mode vectors.

Number of low-frequency modes	5		100	
Normal mode model	ENM	CND	ENM	CND
Apo				
1USG	70.36	70.20	74.77	77.57
4AKE	62.69	66.82	76.67	77.06
1SW5	59.70	56.20	67.38	69.80
1K5H	9.57	8.26	40.34	38.31
1Y3Q	64.10	64.88	74.92	76.36
1GUD	55.88	63.48	69.43	71.18
1RF5	49.98	52.45	61.09	60.88
1ZA1	20.38	20.46	40.83	40.14
1HOO	16.50	19.80	37.78	40.14
1TJD	24.83	29.08	41.51	47.24
1JEJ	41.12	44.02	56.67	57.45
1CA2	3.15	0.91	11.05	5.00
1KPA	1.69	4.08	9.71	11.97
Holo				
1USI	43.27	51.76	61.07	65.36
1AKE	21.41	28.66	53.81	60.96
1SW2	44.46	37.66	56.78	60.90
1Q0Q	9.72	7.60	24.35	33.26
1Y3N	49.56	57.18	66.82	72.95
1RPJ	40.08	37.06	56.91	61.06
1RF4	31.62	25.84	50.91	53.89
1Q95	8.56	10.55	37.31	38.80
1CG0	10.59	12.44	22.73	27.17
1EEJ	26.79	30.46	46.77	48.41
1JG6	34.29	36.37	48.57	52.95
1CIM	0.65	0.93	10.24	5.86
1KPE	2.36	4.37	15.83	11.97

a The coverage of conformational change is obtained from 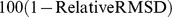. Its higher value indicates better fitting to the conformational change.

It is interesting to note that larger conformational changes are better fitted by CND or ENM normal modes. For example, more than 70% of the conformational changes in 1USG-1USI apo-holo pair (7.32 Å) or in 4AKE-1AKE apo-holo pair (7.19 Å) are covered by the first 100 CND or ENM normal modes (Table 2). On the other hand, small conformational changes are harder to fit by CND or ENM normal modes. For example, less than 12% of the conformational changes in 1CA2-1CIM (0.64 Å) and 1KPA-1KPE (0.53 Å) pairs are covered by the first 100 CND or ENM normal modes.

### 4) Suppression of Fluctuation of Exposed Atoms

**Figure 6:**
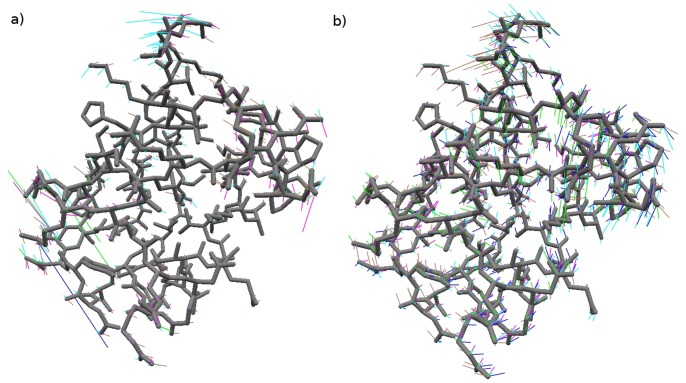
Examples of surface fluctuations. Five lowest-frequency normal modes of Ubiquitin from ENM (a) and CND (b). The atomic displacements from modes 1 to 5 are shown in blue, brown, cyan, green and magenta, respectively. The Ubiquitin structure is shown in the gray stick model. Note the extremely large fluctuations of a small number of surface atoms in ENM modes (a). Such behavior is absent from CND modes (b).

One of the drawbacks of ENM is that it often yields extremely large fluctuations of a small number of the surface or exposed atoms, presumably due to the lack of protein-solvent interactions (e.g., Figure 6a). In CND protein-solvent interactions are implicitly taken into account through the contact number restraints. As a result, the extreme fluctuations of surface atoms are indeed suppressed (e.g., Figure 6b).

**Figure 7:**
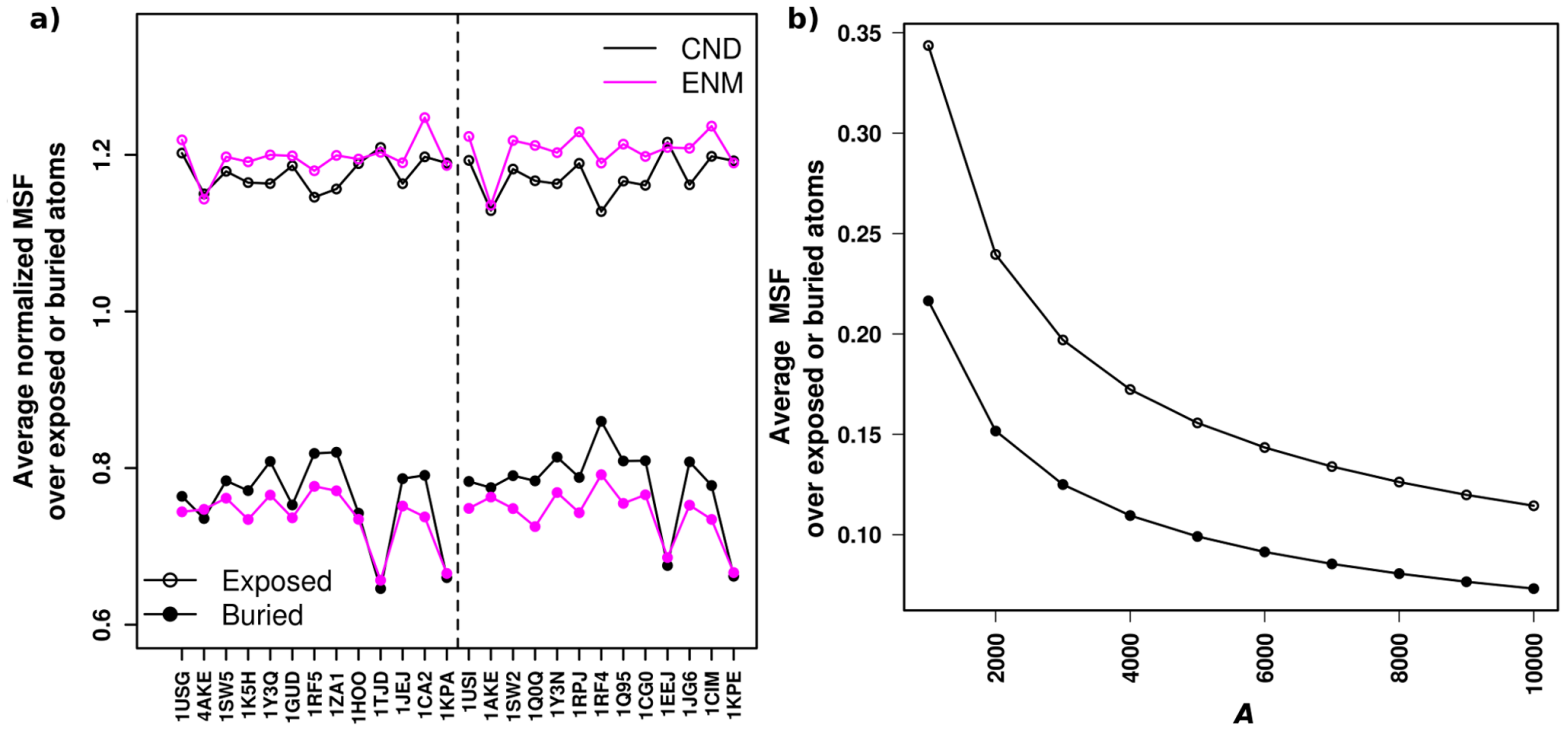
Suppression of surface fluctuations in CND. a) Average normalized MSF over exposed and buried atoms for 26 structures in our data set. The exposed and buried atoms were identified from solvent accessible surface area (ASA), where buried atoms have zero ASA. The MSF of all atoms were normalized so that the average over all atoms is unity in CND and ENM, and therefore the normalized MSF values are unitless. b) Average MSF (in the unit of Å^2^) over exposed and buried atoms by varying parameter *A* of CND for ADK^A^. By increasing *A* the variation of fluctuation between exposed and buried atoms can be decreased, where exposed atoms follow more pronounced changes than buried atoms.

We compared the average fluctuation of exposed atoms (atoms with nonzero accessible surface area (ASA) [19]) in CND and ENM. The normalized MSFs averaged over exposed atoms (see Figure 7a legend) in CND were smaller than that in ENM in majority of the cases (Figure 7a, see also Figure S2). In accordance with this, the normalized MSFs averaged over buried atoms (i.e. atoms with zero ASA) in CND were larger than that obtained from ENM. Overall, the variation of fluctuation between exposed and buried atoms in CND is relatively smaller than that in ENM. This behavior of CND results from the multi-body nature of the contact numbers. Figure 7b showed that by decreasing the parameter *A* of CND the variation of fluctuation between exposed and buried atoms can be increased.

### 5) Fluctuation of CND Modes are Correlated with Experimental Thermal Fluctuations

**Figure 8:**
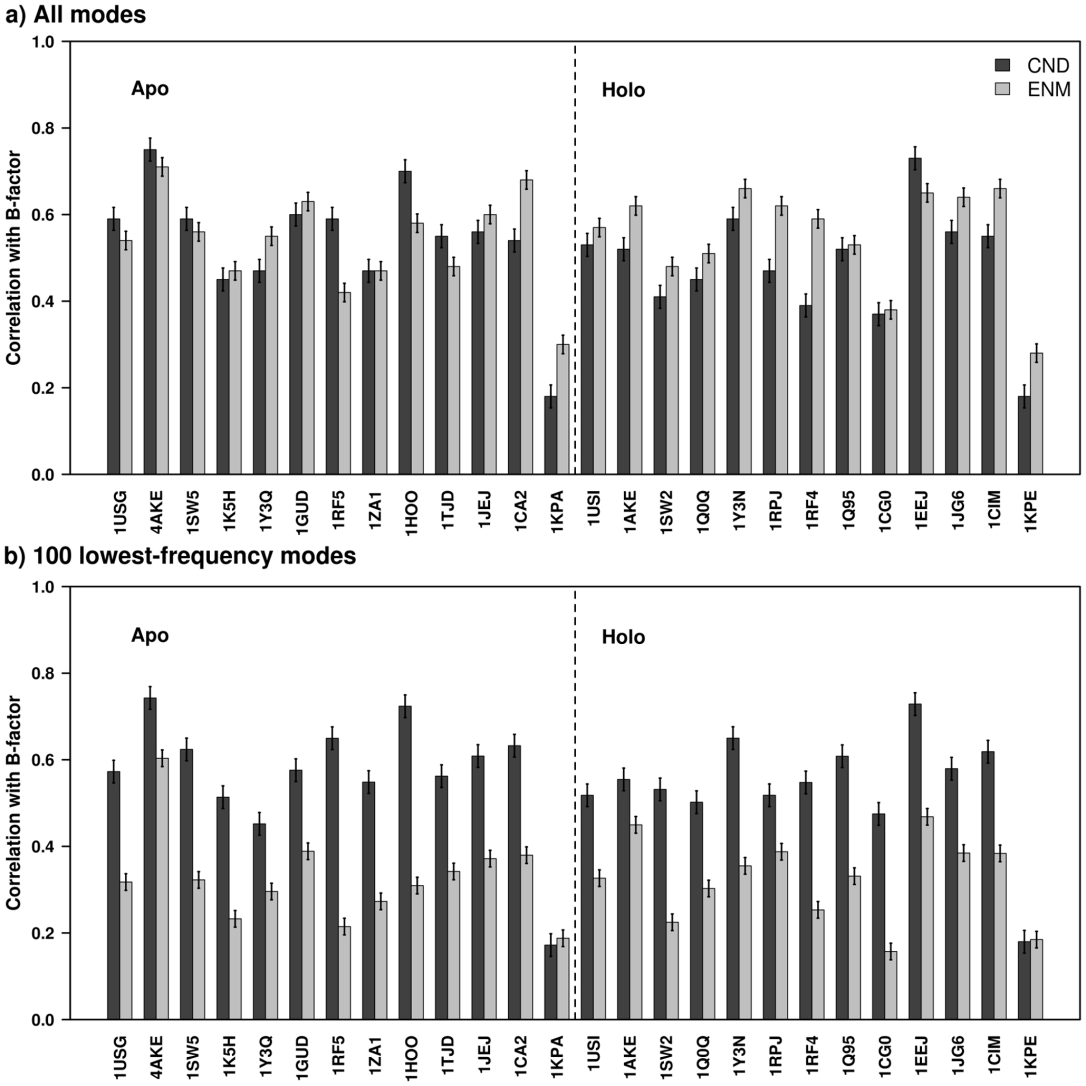
Correlation between MSF and crystallographic B-factors. Correlation between MSF and X-ray crystallographic B-factor for the 26 structures in the data set obtained from CND and ENM. The right and left panels (separated by a dotted-line) include results from apo and holo structures, respectively. The errorbars were calculated using bootstrapping. The MSFs were obtained from all the vibrational modes (in (a)), and first 100 low-frequency modes (in (b)).

The normal mode analysis obtains thermal motion of a protein around its equilibrium configuration. Therefore, the thermal characteristics obtained from such models, i.e. MSF, can be compared with experimental B-factors. For the 26 structures in the current dataset ENM showed slightly better correlations than CND (Figure 8a: in 9 cases they were comparable; 5 cases, CND was better; 12 cases, ENM was better).

By dividing the 26 structures into apo and holo structures we found that in general for holo structures ENM performed better than CND, while for apo structures CND performed slightly better than ENM (Figure 8a). For example, in case of the 1RF5-1RF4 pair of apo-holo structures CND showed better correlation than ENM (0.59 over 0.42) in the apo condition, whereas ENM showed better correlation than CND (0.59 over 0.39) in the holo condition. This suggests that specific non-local interactions as in ENM may play a greater role in more compact (e.g., ligand-bound) structures.

We also compared MSFs of CND and ENM computed using the first 100 low-frequency modes, rather than all the modes, with crystallographic B-factor (Figure 8b). The low-frequency modes of CND showed significantly higher correlation to B-factor than ENM for all the structures, except for human protein kinase C interacting protein 1 (1KPA and 1KPE; these are an apo-holo pair). A comparison of MSFs in CND using the low-frequency modes (the average correlation over all the structures was 0.57) and all the modes (0.54) showed that the correlation with B-factor did not improve significantly (P-value of Students’ t-test was 2.985×10^−1^) by using all the modes. A similar comparison in ENM (0.35 against 0.56) showed that the correlations improved significantly (P-value was 8.104×10^−14^). These results were in accordance with Figure 1, showing the dominance of low-frequency modes in CND in overall dynamics.

## Discussion

The normal mode vibrations of a protein characterize its large-scale motions. Such vibrational motions were successfully used previously to predict the conformational change. Also it has been observed that in many cases normal modes match well to the MD simulation data [10], [20]. In NMA the construction of a network model is crucial to obtain meaningful dynamic characteristics of the protein [9]. In all-atom ENM such a network is obtained by modeling each protein atom as a node and the potential energy of the system depends on the pairwise distance information among all the nodes [4], [9]. This indicates that for an *N*-atom system we need *N* ^2^ number of distance information to restrain the motion of a protein to its native state. This implies that the primary sequence of a protein has to somehow contain *O*(*N^2^*) information to exhibit its function via the dynamic structure. On the other hand if each atom in an *N*-atom system includes only its local structural properties then the number of restraints can be considerably reduced. In fact, specific local structures are known to play a very important role in determining the native structure [21]. In CND we restrained the local structure of a protein molecule by a sum 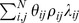 where *θ* was defined with the window size of 1 (i.e. tri-peptide segments). By using this in CND the number of restraints are in the order of *N* (Table S1). Despite the fact that CND uses fewer restraints than ENM we observed that CND captures native protein dynamics well. We have observed in many respects the results obtained under these two models are comparable.

Let us consider the similarities and differences between CND and ENM. As far as local interactions are concerned, the two models are essentially identical. In fact, ENM is a limiting case of CND when the window size covers the complete polypeptide chain (see theory section discussing ENM, eqns. 23, 24). As for the non-local interactions, ENM requires all specific pairwise interactions in the same manner as local interactions, whereas CND requires only semi-specific interactions in terms of contact numbers. In previous studies it was indicated that contact numbers included significant amount of information about the native structure [22]–[24]. Our study further indicates that the non-local contact numbers also include significant amount of information about the native dynamics. Another difference between CND and ENM is the way of imposing native restraints. While ENM imposes native restraints as harmonic potential, CND imposes native restraints by the Lagrange multipliers in conjunction with autonomous terms. This formulation of CND makes it an extensible model. For any fine-tuning of the energy function one may add more terms in the autonomous part and the corresponding Lagrange multipliers. One distinguishing feature of CND is the autonomous diffusion term (the second term of right hand side of eq. (8)). This term penalizes a large difference in contact numbers between locally contacting atoms; thus these atoms tend to have similar contact numbers, whereas atoms that are far apart may have very different contact numbers. In this sense, CND models phase separation of high and low contact numbers, or of hydrophobic and hydrophilic residues.

**Figure 9:**
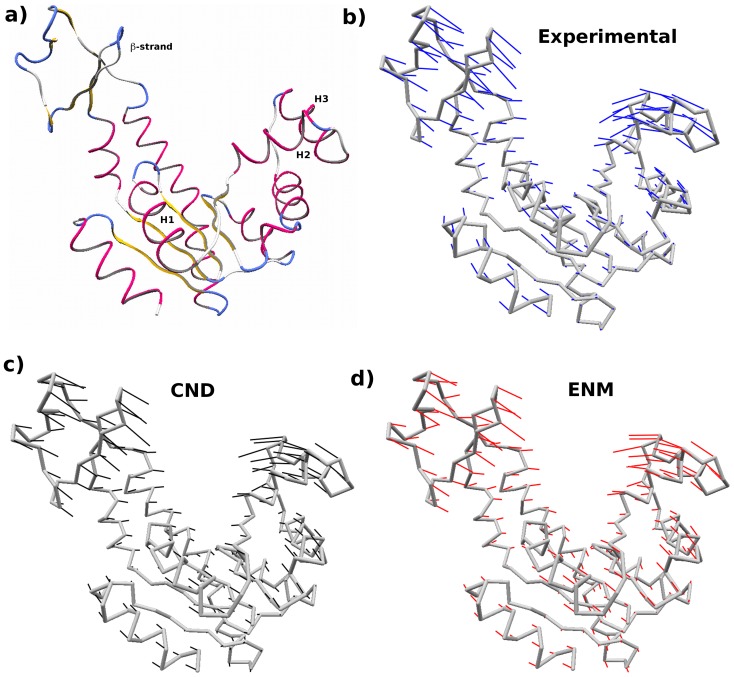
Experimental conformational change expressed in terms of linear combination of normal modes. a) Tube representation of ADK^a^ structure. The helices (H1, H2 and H3) and β-strand that show large-scale motion during the apo to holo conformational change were annotated. The helices are numbered from the amino-acid sequence, where ‘H1′ represent the first helix from the N-terminal. b) The apo-holo conformational change is shown by atomic displacement vectors in blue. c,d) The atomic displacement vectors (in black and red, respectively) were obtained from least-square fitting of the first 5 low-frequency modes to the apo-holo conformational change. In (c) and (d) normal modes in CND and ENM were used respectively.

One of the important findings in the present study was good fitting of conformational changes by CND normal modes (Figure 4, 5). Figure 4 showed comparable performance of CND and ENM in the cumulative least-square fitting of the first 100 low-frequency modes of ADK^A^. This is further exemplified in Figure 9. The experimentally observed conformational change from apo to holo structures (Figure 9b) is nearly identical to the conformational changes obtained from the best-fitting linear combination of the 5 lowest-frequency normal modes vectors in CND (Figure 9c) and ENM (Figure 9d). In particular, closing of helices and β-strands in two distal lobes are similar in Figure 9b–d.

**Figure 10:**
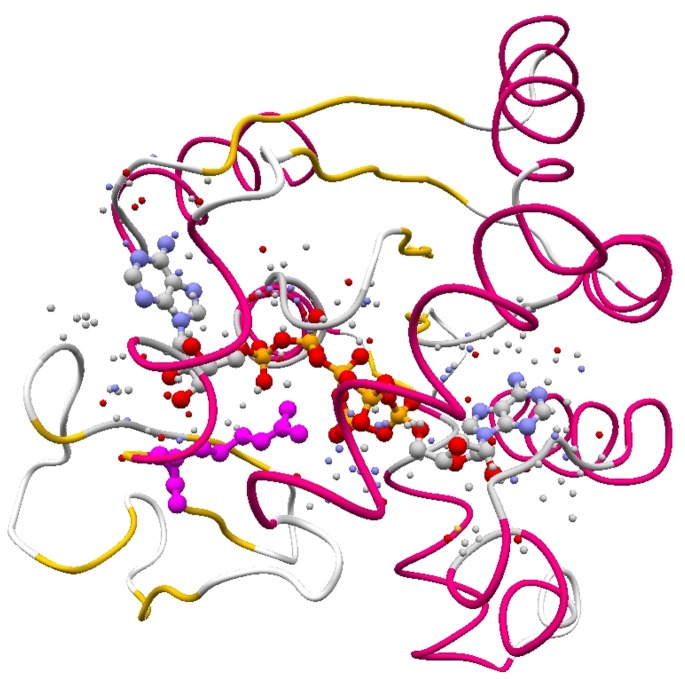
Specific protein-ligand interaction in holo ADK. The protein atoms that interact with ligand bis(adenosine)-5′-pentaphosphate (in ball-and-stick) in holo ADK (in tube representation) are shown in cpk color scheme and in spacefill representation. The side-chain amide nitrogen of Arg123 residue (shown in ball-and-stick and in magenta) is buried (zero accessible surface area) and shows maximum fluctuation over all the buried atoms in holo ADK using CND. The interacting atoms of the protein are obtained from a distance threshold of 5 Å. From ENM we obtained that the normalized MSF (see Figure 6 legend) averaged over the interacting atoms (0.75) were significantly lower (P-value 9.22×10^−61^ by t-test) than averaged over the non-interacting atoms (1.032).

We observed that CND suppressed motion of the exposed atoms. The variation of normalized fluctuation between exposed and buried atoms is observed to be less in CND than in ENM (Figure 7a). Such an analysis indicated that the magnitudes of fluctuation of buried atom relative to the exposed atom from CND is greater than that obtained from ENM. For holo cases we observed that such significant motion of the buried atoms is due to the exclusion of ligand molecule in NMA or no consideration of hydrogen-bond network. In 3 out of 13 holo structures we observed that such highly fluctuating buried atoms in CND interact with the ligand molecules, which are not included in NMA of the holo structures. One such example of highly flexible buried atom is shown in Figure 10, where the side-chain amide nitrogen of Arg123 in holo ADK (1AKE) shows high MSF and also interacts with the ligand molecule. Such a high fluctuation is not observed in ENM. Moreover, the atoms that specifically interact with the ligand molecule show significantly lower fluctuation than the non-interacting atoms in ENM. Note that, in ENM all the non-local interactions are specific, whereas in CND those interactions are semi-specific. Do specific interactions among the protein atoms near its active site dictate protein-ligand interaction specificity? Such an analysis may provide valuable information regarding the mechanism of the binding process and would be an interesting subject for future studies.

The thermal characteristics in CND and ENM can be obtained from all the vibrational modes or only the low-frequency modes. When all the vibrational modes were used the MSF from ENM correlated with the B-factors slightly better than CND. When only the first 100 low-frequency modes were used CND correlated much better than ENM (Figure 8). The correlations with B-factor saturated by first 100 low-frequency modes in CND but not in ENM (from the comparison between Figure 8a and b). In Figure 8, 1KPA and 1KPE were the exceptions those showed significantly low correlation with the B-factor. This can be explained on the basis of dimeric structure of 1KPA or 1KPE. In the present analysis we performed NMA of the chain B of those structures (Table 1), and that completely disregards any inter-subunit contacts. The correlation of MSF (from all the modes) of chain B with B-factor significantly improved from 0.18 to 0.55 by NMA of the whole complex (considering chain A and B together); the corresponding correlation in ENM improved from 0.30 to 0.63.

The correlation of MSF from CND with B-factor depends on the parameters of CND (Figure S1). For example, the correlation with B-factor increased with increasing parameter A of CND (Figure S3). However, when parameter B was set to zero a negative correlation (−0.25) was observed, which indicated the importance of semi-specific non-local interaction.

There exist a number of models for NMA that use stability of protein local structure or contact numbers. For example, the chemical network model introduced by Kondrashov *et al.* [25] classifies inter-residue connections into different types of Hookean springs depending on the residue types. This model successfully predicted crystallographic B-factors by separating bonded and non-bonded interactions in the Hessian matrix. Ming and Brüschweiler [26] introduced the reorientational contact-weighted ENM to predict experimental N–H bond order parameters. A different approach was taken by Halle [27], who related atomic mean square displacements to the reciprocal of local density of an atom. In his work Halle approximated that an atom undergoes harmonic fluctuation under a potential of mean force (‘local density model’). Later, Li and Brüschweiler [28] introduced an all-atom contact model by the combination of the reorientational contact-weighted ENM and the local density model. All of the above models were introduced mainly to predict X-ray crystallographic B-factor. However, CND model is not aimed to predict only B-factors. Rather, it is aimed to obtain functionally relevant collective motion of a protein. Atilgan *et al.* [29] also showed that by separating the Hessian matrix into ‘essential’ (including specific contacts) and ‘residual’ (including non-specific contacts) parts the collective motions of a protein could be identified only from the essential part. In their study the ‘essential’ part included both local and non-local contacts. However, in the present study we separated local and non-local contacts on the basis of chemical structure.

### Conclusion

We introduced and evaluated a new model, the contact number diffusion model, to understand collective dynamics of protein structures. This model aims to model local phase separation between hydrophilic and hydrophobic components in native protein structures. While this “phase separation” (i.e., “hydrophobic in, hydrophilic out”) is believed to be an important determinant of the protein structure, it was not possible with ENM to relate this principle to protein dynamics, and hence protein function. However, rather than treating hydrophobicity directly, we have used the contact number which is dually related to hydrophobicity [14], [15]. Most importantly, the result of this study has shown that CND can yield dynamic characteristics comparable to ENM in spite of much fewer restraints than ENM. Additional benefits of CND over ENM are reduced surface fluctuations (Figure 6) and more collective motions (Figure 1). The dynamic features obtained from our model correlated well with the MD simulation result (Figure 2, 3). Moreover, low-frequency modes of CND matched apo-holo conformational changes (Figure 4, 5) and correlated well with B-factors (Figure 7). The CND model generalizes ENM, where the latter is a limiting case of the former (eq. (18B)). In summary, the results presented here suggest non-local or long-range interactions need not to be fully specific for reproducing native protein dynamics when the solvent effect is taken into account.

## Materials and Methods

### 1) Data Set of Protein Structures

We took 13 pair of protein structures [30]–[49] (Table 1) from a previous work by Wako and Endo [13], where each pair included an apo (ligand unbound) and a holo (ligand bound) structures. In all the apo-holo pairs, the numbers of atoms in the apo and holo structures were identical. In most cases the data set included an apo structure that shows large-scale domain motions upon ligand binding (Table 1) [50]. For three pairs of apo-holo structures ligand binding involves side-chain conformational changes [51]. Moreover, 6 of the 13 pairs were used to predict holo structures from the apo structures in a docking benchmark study [52].

To compare surface fluctuations in CND and ENM we have used all the above 26 structures and additionally Ubiquitin structure (1UBQ [53], chain A, residues 1 to 72).

### 2) Determining CND Parameters

In CND there are four free parameters, *viz*. *A*, *B*, *w* and *σ*. A few initial runs indicated that *A* need to be three orders of magnitude larger than *B* in order to fit apo-holo conformational change. To find an optimum set of parameters we fixed *B* at 1 unit and varied *A* as 1000, 5000 or 10000 unit, *w* as 1, 3 or 5 and *σ* as 1, 2 or 3, and obtained normal modes from CND of apo ‘adenylate kinase’ (PDB ID: 4AKE, referred to as ADK^A^ in the following, where superscript ‘A’ indicates apo structure), apo ‘L-Leu binding protein’ (1USG) and apo ‘human protein kinase C interacting protein 1′ (1KPA) (Figure S1). We chose above three proteins because (1) the 1USG-1USI pair shows largest conformational change (Table 1), (2) the 4AKE-1AKE pair is one of the standard model systems to analyze a large conformational change, and (3) the 1KPA-1KPE pair shows very small conformational change to which ENM of the apo structure hardly fits. Apart from fitting to the apo-holo conformational changes, the MSF obtained from NMA of different runs were correlated with the B-factor of the structures. The results of this parameter search were compared to that from ENM. We observed from Figure S1 that better results are obtained at highest *A* (10000 unit) and when *σ* is 2 or 3 and *w* is 1. Therefore, we set *w* = 1 and *σ* = 2 Å to perform NMA using CND of all the 26 structures (Table 1). Note that, the value of *d_cut_* was set at 5 Å. The value of *A = *10000 may appear very large compared to *B = *1. Nevertheless, the contribution of terms involving *A* to the Hessian is limited to the band-diagonal elements (Eq. 14D), and the only contribution to the other off-band-diagonal elements comes from terms with *B* and the number of off-band-diagonal elements are far greater than the number of band-diagonal elements. Therefore, however small the value of *B* (as long as it is not zero), the contact number diffusion term imposes a non-negligible effect on the dynamics.

### 3) Normal Mode Analysis

We performed NMA of all-atom system using CND and ENM. The source codes to perform NMA of CND were written in the R programming language [54] and that of ENM was written in C [12], [55]. In CND and ENM we set *d_cut_* to 5 Å.

We diagonalized mass-weighted Hessian matrix to obtain all non-zero eigenvectors (3*N*-6 in number). The DSYEVR routine of LAPACK was used for diagonalization [56]. The molecular figures were obtained by using *j*V [57], [58] for which the atomic displacement vectors were prepared by a combination of perl and R scripts.

### 4) Molecular Dynamics Simulation

ADK^A^ (4AKE) was subjected to a 12 ns NVT molecular dynamics simulation (time step 1 fs) in explicit water using the GROMACS program [59]. The system was set up in the following way. The Amber99SB force field was used for protein [60]. Initially the protein molecule was immersed in a 55×65×75 Å^3^ simulation box containing 7322 TIP3P water molecules [61] with periodic boundary condition. The particle mesh Ewald method was used for electrostatic interactions with 12 Å cutoff and a dumping factor 0.26 Å^−1^. Adding 24 Na^+^ and 20 Cl^-^ ions neutralized four additional charges of the protein and the final concentrations of ions were 0.15M. The final system consisted of 25351 atoms. Such a system was energy minimized in two steps. First the system was subjected to the conjugate gradient energy minimization with positional restraints on heavy atoms until the maximum force became less than 100 kJ/mol/nm. Further conjugate gradient minimization was applied without positional restraints (with the same tolerance). Before production run the system was subjected to 100 ps NPT simulation (time step 0.5 fs) at P = 1 atm and T = 300 K to equilibrate against Berendsen barostat [62], where positions of the heavy atoms were restrained to the initial structure of the simulation. After equilibration the system size becomes 54.0×63.8×73.7 Å^3^. In the production run we saved 12000 snapshots in total for 12 ns. Here, the covalent bonds between hydrogen atoms and heavy atoms were constrained with the LINCS method [63]. For the analysis of the trajectory we discarded the first 2 ns of the trajectory.

### 5) Least-square Fitting of Normal Modes to Conformational Changes

To define a conformational change between apo and holo structures we superimposed the former to the latter. The difference between the superimposed coordinates of apo and holo structures defines the conformational change (e.g. *Y^AH^* represents a vector of mass-weighted conformational change from apo to holo). We approximated the normalized conformational change (i.e. 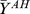 or 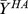, where 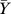 is obtained by normalizing *Y*) by a linear combinations of the normal modes [13]. For example, an apo-holo conformational change is approximated as,
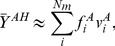
(23A)where 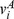 is the *i*-th normal mode of the apo structure, 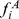 is its coefficient, and *N_m_* is the number of normal modes considered in the fitting. In a similar way, we approximate holo-apo conformational change by
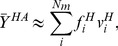
(23B)where, 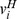 is the i-th normal mode of the holo structure and 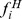 is its coefficient. The above procedure is similar to the least-square fitting of the conformational change by a set of normal mode vectors discussed in the reference [13].

We performed the above least-square fitting by sets of normal mode vectors to the conformational change cumulatively (i.e. by varying *N_m_* from 1 to 100) obtained from the CND and ENM. We evaluate the performance of such fitting by 
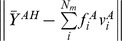
 or 
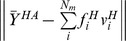
, which we call relative RMSD in the following. This quantity is bounded between 1 (i.e. complete failure in fitting) and 0 (i.e. complete fitting).

We also analyzed MD trajectory by principal component analysis [64], and fitted 30 lowest-frequency principal components of ADK^A^ by cumulative addition of 100 low-frequency CND or ENM modes.

## Supporting Information

Figure S1 **Determination of CND parameters.** We searched for the optimal set of parameters (*A*, *w* and *σ*) by varying them for the structures (4AKE, 1USG, and 1KPA). Among these structures the former two show a large conformational change between the apo and holo conformations and 1KPA includes small structural change between apo to holo. We compared the maximum and minimum relative RMSD obtained by fitting the conformational change to the first 100 normal modes (left panel, solid circles indicate maximum relative RMSD by using only the first mode, open circles minimum relative RMSD by using all the 100 modes). In the right panel we compared correlation between the B-factor and the MSF obtained from all the normal modes. In all the figures the points shown in red are obtained from NMA of ENM.
(DOC)

Figure S2 **Comparison of maximum atomic fluctuation in CND and ENM.** Maximum of the normalized MSF over exposed and buried atoms for 26 structures in our data set (Table 1, main text). The MSF of all atoms were normalized so that the average over all atoms was unity in CND and ENM.
(DOC)

Figure S3 **Influence of parameter** ***A*** **of CND to the correlation between B-factor and MSF.** Correlation between MSF and B-factor with increasing values of parameter *A* of CND for ADK^A^. The horizontal dotted line (in magenta) indicates correlation obtained from ENM of ADK^A^.
(DOC)

Table S1 Number of restraints used on the apo structure in CND model.
(DOC)
