# Supplementary material for: Specific Non-Local Interactions Are Not Necessary for Recovering Native Protein Dynamics
Source: PLoS One. 2014 Mar 13;9(3):e91347. doi: 10.1371/journal.pone.0091347 (PMC3953337; doi:10.1371/journal.pone.0091347)
Supplement: Figure S1 — Determination of CND parameters. We searched for the optimal set of parameters (A, w and σ) by varying them for the structures (4AKE, 1USG, and 1KPA). Among these structures the former two show a large conformational change between the apo and holo conformations and 1KPA includes small structural change between apo to holo. We compared the maximum and minimum relative RMSD obtained by fitting the conformational change to the first 100 normal modes (left panel, solid circles indicate maximum relative RMSD by using only the first mode, open circles minimum relative RMSD by using all the 100 modes). In the right panel we compared correlation between the B-factor and the MSF obtained from all the normal modes. In all the figures the points shown in red are obtained from NMA of ENM. (DOC) [file pone.0091347.s001.doc]

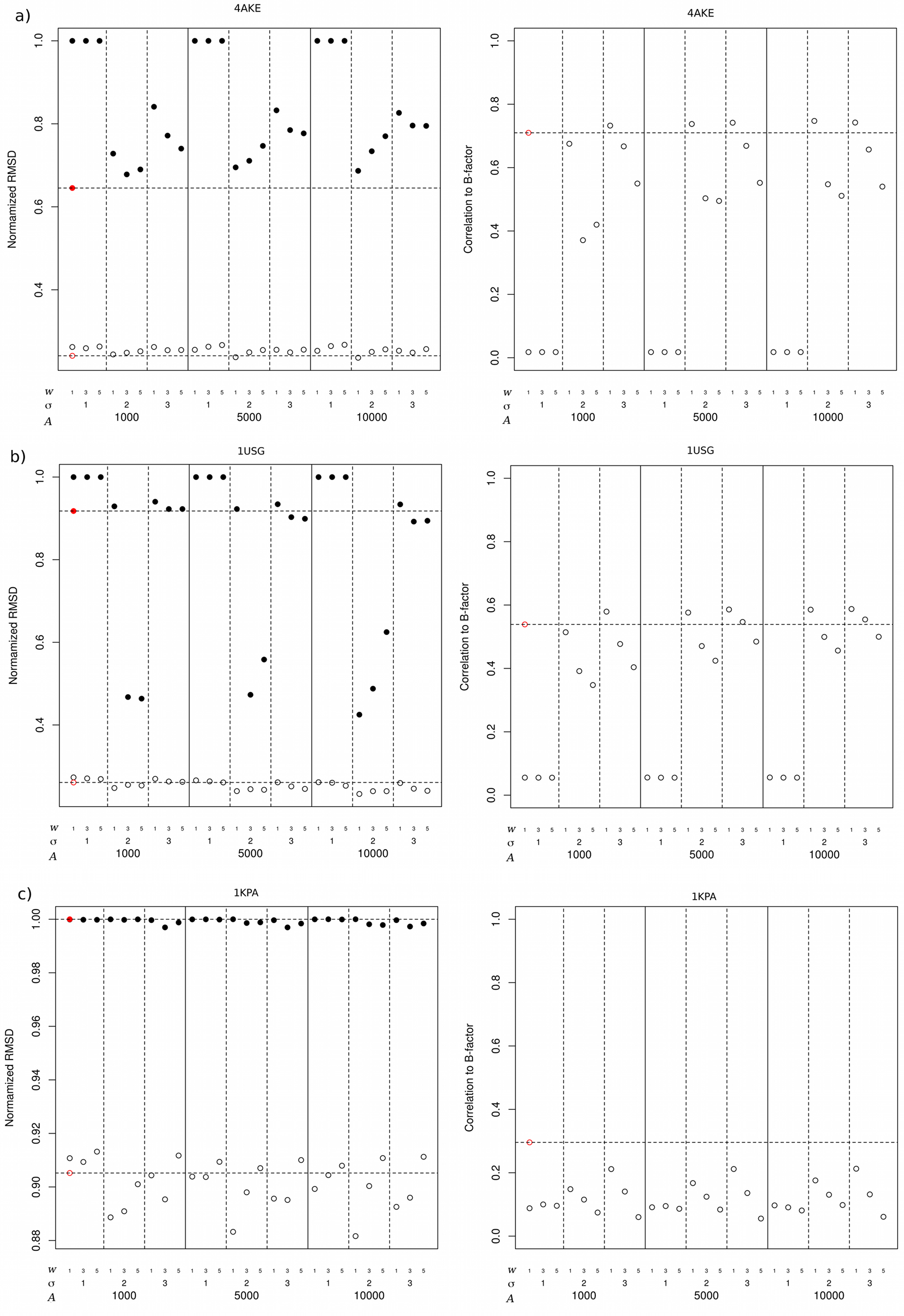


**Supporting Figure S1: Determination of CND parameters:** We searched for the optimal set of parameters (*A*, *w* and *σ*) by varying them for the structures (4AKE, 1USG, and 1KPA). Among these structures the former two show a large conformational change between the apo and holo conformations and 1KPA includes smsall structural change between apo to holo. We compared the maximum and minimum relative RMSD obtained by fitting the conformational change to the first 100 normal modes (left panel, solid circles indicate maximum relative RMSD by using only the first mode, open circles minimum relative RMSD by using all the 100 modes). In the right panel we compared correlation between the B-factor and the MSF obtained from all the normal modes. In all the figures the points shown in red are obtained from NMA of ENM.
