# Supplementary material for: Specific Non-Local Interactions Are Not Necessary for Recovering Native Protein Dynamics
Source: PLoS One. 2014 Mar 13;9(3):e91347. doi: 10.1371/journal.pone.0091347 (PMC3953337; doi:10.1371/journal.pone.0091347)
Supplement: Figure S2 — Comparison of maximum atomic fluctuation in CND and ENM. Maximum of the normalized MSF over exposed and buried atoms for 26 structures in our data set (Table 1, main text). The MSF of all atoms were normalized so that the average over all atoms was unity in CND and ENM. (DOC) [file pone.0091347.s002.doc]

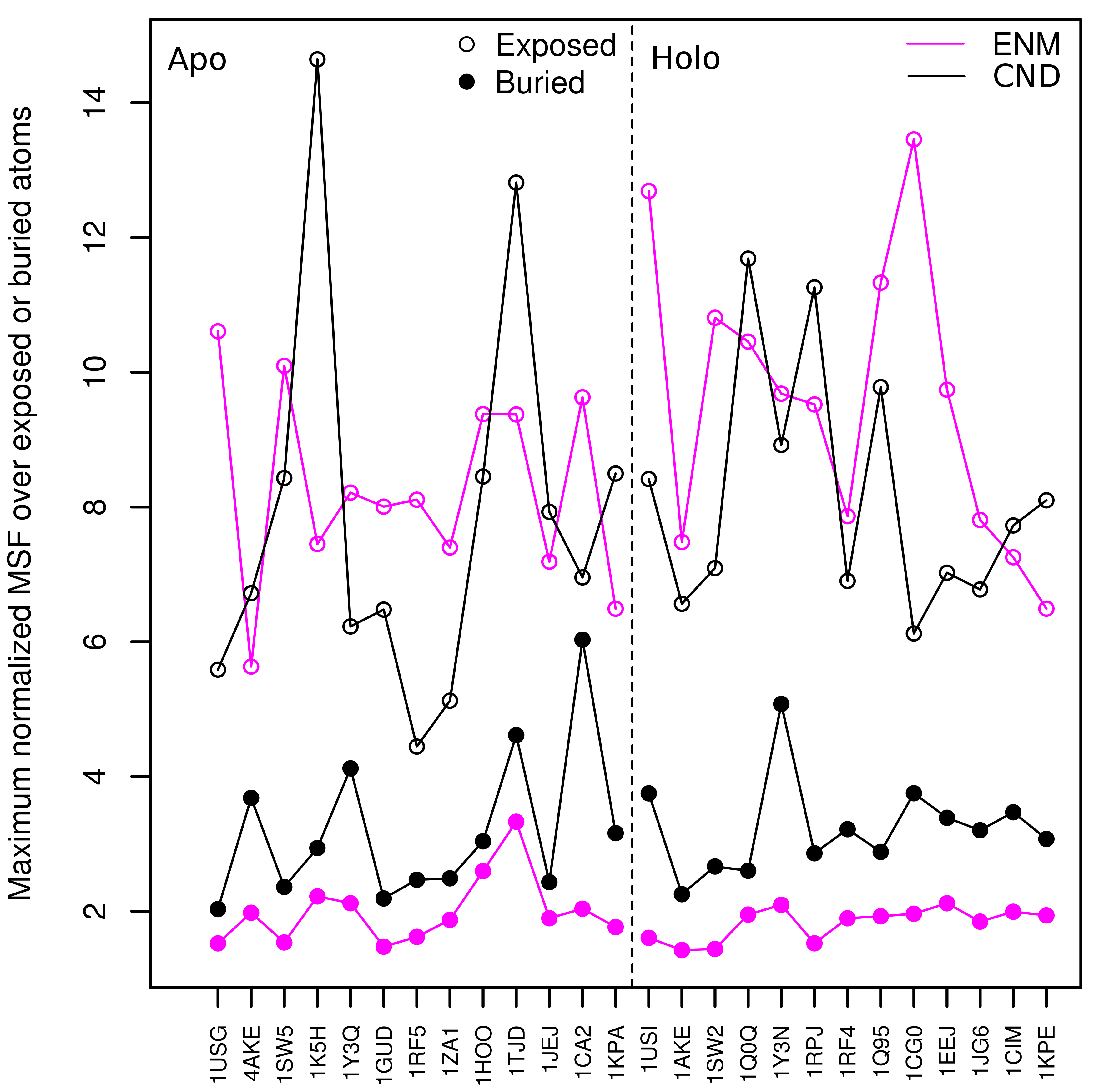


**Supporting Figure S2. Comparison of maximum atomic fluctuation in CND and ENM:** Maximum of the normalized MSF over exposed and buried atoms for 26 structures in our data set (Table 1, main text). The MSF of all atoms were normalized so that the average over all atoms was unity in CND and ENM.
