# Supplementary material for: Specific Non-Local Interactions Are Not Necessary for Recovering Native Protein Dynamics
Source: PLoS One. 2014 Mar 13;9(3):e91347. doi: 10.1371/journal.pone.0091347 (PMC3953337; doi:10.1371/journal.pone.0091347)
Supplement: Figure S3 — Influence of parameter A of CND to the correlation between B-factor and MSF. Correlation between MSF and B-factor with increasing values of parameter A of CND for ADKA. The horizontal dotted line (in magenta) indicates correlation obtained from ENM of ADKA. (DOC) [file pone.0091347.s003.doc]

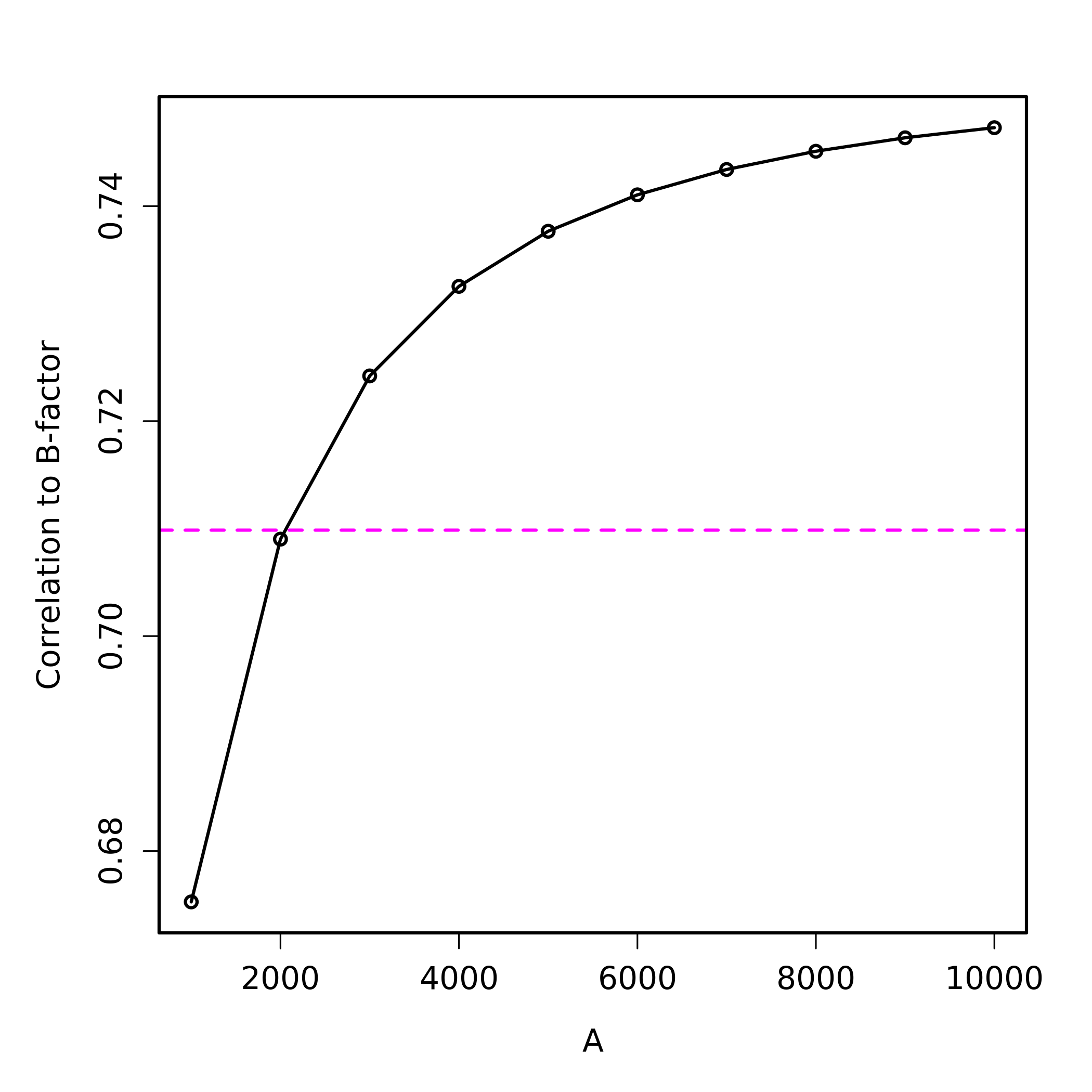


**Supporting Figure S3. Influence of parameter *A* of CND to the correlation between B-factor and MSF:** Correlation between MSF and B-factor with increasing values of parameter *A* of CND for ADK^A^. The horizontal dotted line (in magenta) indicates correlation obtained from ENM of ADK^A^.
