# Supplementary material for: Specific Non-Local Interactions Are Not Necessary for Recovering Native Protein Dynamics
Source: PLoS One. 2014 Mar 13;9(3):e91347. doi: 10.1371/journal.pone.0091347 (PMC3953337; doi:10.1371/journal.pone.0091347)
Supplement: Table S1 — Number of restraints used on the apo structure in CND model. (DOC) [file pone.0091347.s004.doc]

**Supporting Table S1:**

**Number of restraints used on the apo structure in CND model**

| PDB ID | Number of atoms | Fraction of number of local restrains over number of atoms^a^ |
| --- | --- | --- |
| 1USG | 2596 | 12.09 |
| 4AKE | 1656 | 12.33 |
| 1SW5 | 2161 | 12.67 |
| 1K5H | 3030 | 12.19 |
| 1Y3Q | 4025 | 13.09 |
| 1GUD | 2133 | 11.85 |
| 1RF5 | 3212 | 12.00 |
| 1ZA1 | 2415 | 12.40 |
| 1HOO | 3321 | 12.35 |
| 1TJD | 1640 | 12.11 |
| 1JEJ | 2869 | 12.96 |
| 1CA2 | 2039 | 12.70 |
| 1KPA | 874 | 12.40 |

^a^ Fraction of local restraints were obtained from$\left(\sum_{i,j\neq i}^{N} \theta_{ij}+\sum_{i}^{N} \theta_{ii}\right)/N$.
